# A mobile robot safe planner for multiple tasks in human-shared environments

**DOI:** 10.1371/journal.pone.0324534

**Published:** 2025-06-12

**Authors:** Jian Mi, Xianbo Zhang, Zhongjie Long, Jun Wang, Wei Xu, Yue Xu, Shejun Deng

**Affiliations:** 1 Department of Transport Engineering, College of Architecture Science and Engineering, Yangzhou University, Yangzhou, Jiangsu, China; 2 College of Mechanical and Electrical Engineering, and Key Laboratory of the Ministry of Education for Modern Measurement and Control Technology, Beijing Information Science & Technology University, Beijing, China; 3 College of Information Science and Technology, Beijing University of Chemical Technology, Beijing, China; 4 Smart Lab of Innovation, Sinotrans Innovation and Technology Co., Ltd, Beijing, China; Shanghai Jiao Tong University - Xuhui Campus, CHINA

## Abstract

Various approaches have been studied to solve the path planning problem of a mobile robot designing with multiple tasks. However, safe operation for a mobile robot in dynamic environments remains a challenging problem. This paper focuses on safe path planning for a mobile robot executing multiple tasks in an environment with randomly moving humans. To plan a safe path and achieve high task success rate, a safe planner is developed where a double-layer finite state automaton (FSA)-based risk search (FSARS) method considering environmental risks is proposed. The low-level of FSARS is a novel safe approach to prioritize a safe path rather than merely seeking the shortest path in dynamic environments. Meanwhile, the high-level implements a safety-first search structure utilizing FSA transitions. This structure aims to generating optimal paths while multitasking, avoiding collisions with humans moving completely randomly at the planning level instead of aiming at real-time collision avoidance. FSARS is verified through a series of comparative simulations involving seven types of environmental settings, each with distinct task number, grid size, and human number. We evaluate FSARS based on several metrics, including conflict number, conflict distribution, task success rate, reward, and computational time. Compared with the reinforcement learning method, FSARS reduces the average conflict by 65.4% and improves the task success rate by 34.4%. Simulation results demonstrate the effectiveness of FSARS with the lowest collisions and the highest success rate compared with classic approaches.

## 1 Introduction

### 1.1 Motivation

Over the past few years, autonomous mobile robots have been extensively employed in automatic factories [1], logistic centers [2], warehouses [3], manufacturing systems [4, 5], and improve efficiency greatly in transport, pick up and delivery, such as Amazon Kiva robot. Nowadays, the demand for autonomous mobile robots collaborating with humans is increasing rapidly. Avoiding conflicts between robots and humans becomes an urgent problem to be solved for controlling robots to execute multiple tasks in human-shared environments.

**Fig 1:**
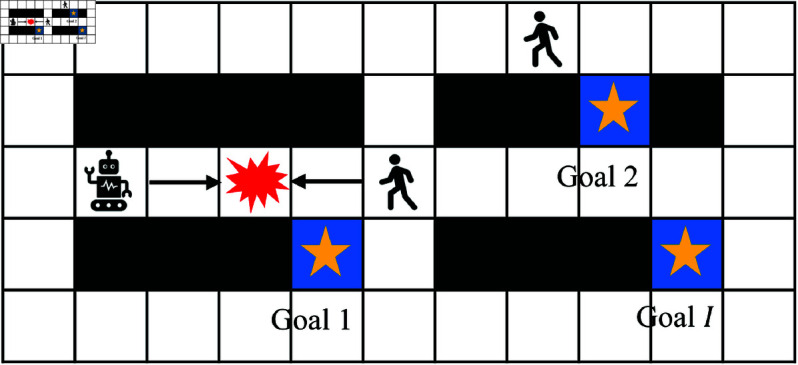
Warehouse domain: a mobile robot works in human-shared environments.

Path planning, one of the important problems of controlling a mobile robot vehicle in dynamic environments, is to generate an optimal conflict-free path from its start location to goal locations more than just shortest path planning (SPP) [6, 7]. Thus far, various methods have been developed to avoiding moving obstacles and real-time path planning algorithms [8] are the common solutions to dynamic environments for safe operation. Unfortunately, those real-time path planning methods need expensive computation cost by continue re-planning and cannot avoid conflicts at the planning level. In addition, the real-time methods are not suitable for those cases as warehouses as Fig 1 shows where a mobile robot has no choice but to escape from human when they encounter, especially for a mobile robot assigned multiple tasks with a given time-step budget. Ensuring safety, encompassing both humans and mobile robots, in such scenarios is a crucial task. As is all known, the real-time planning for each time-step is the safest way for conflict avoidance [9]. However, it’s quite difficult for realization under the cases of warehouses, logistic centers, and automatic factories, not applicable in real-world.

Task controlling is another import problem of a mobile robot vehicle assigned with multiple tasks that the robot has to visit different goal positions to perform specified tasks. We need to find out the optimal visiting order of performing multiple tasks which is famous as vehicle routing problem (VRP) [10]. However, the path between any two goals are unknown. Apparently, the path planning and task controlling problems are deeply and mutually affected. What’s more, both of them are highly influenced by the moving humans.

In human-shared environments, safe operation of a mobile robot vehicle is the most basic and important. In the meanwhile, achieving high task success rate for improving the efficiency of warehouses, automatic factories, etc., is equally significant. Motivated by this, we propose a mobile robot safe planner solving the path planning and task controlling problems where a double layer finite state automaton based risk search method is developed to plan a global safe path for a robot performing multiple tasks in an environment with randomly moving humans. Compared with existing methods, the proposed method solves the conflicts with humans at the planning level.

### 1.2 Related works

In path planning, traditionally, avoidance of obstacles is the primary requirement for mobile robots. Path planning in static environment has already been well studied and numerous efficient algorithms has been developed, such as A^*^ [11, 12], D^*^ [13, 14], Dhouib-Matrix-SPP (DM-SPP) [15] and its variants DM-SPP-4 [16], DM-SPP-24 [17]. However, as is widely known, they are efficient in finding the shortest path in static environments, but struggle to address uncertainties in dynamic environments. Reinforcement learning (RL) [18, 19] and Monte Carlo tree search (MCTS) [20, 21] have been proposed for path planning and task assignment. They are effective in both static and dynamic settings. Unfortunately, these approaches typically require extensive learning cost to derive an optimal policy by repeating trials. What’s more, they cannot provide any guarantees for the learned policy, even in static environments.

Those classic algorithms do not address uncertain factors well, especially obstacles that are unpredictable, such as human operators. When an operator suddenly appears in front of a mobile robot, the operator has to stop proactively and even avoid the robot’s upcoming path to give priority to the robot, ensuring the safety of an operator. Afterwards, more robust methods [22–25] have been presented for human-robot coexistence environments. For instance, a novel approach to collision avoidance for mobile robots based on human-robot interaction was proposed in [26]. The robot will notify the human about its existence via voice messages. After a certain distance, the human needs to give a feedback to the robot to accomplish the interaction. Zeng *et al*. presented a virtual force field-based mobile robot navigation algorithm [27], in which both active and critical regions are used to deal with the uncertainty of human motion. Then, the region sizes based on worst-case avoidance conditions were calculated for the robot navigation. Although they have shown promising results, the abovementioned methods consider a human as a fixed subject in the physical workspace.

The hybrid method combining global path planning (GPP) and local path planning (LPP) [28] is widely applied in navigation where LPP is used for real-time obstacle avoidance. This scheme is widely utilized in the field of autonomous driving [29]. Strictly speaking, it is a real-time planning system and the re-planning mechanism is invoked once it encounters an unexpected obstacle. The big advantage is that it provides the safety. However, it won’t consider the future risk or uncertainties in the planning level. In a scenario of warehouse where the mobile robot works together with random moving humans, usually, a mobile robot needs to accomplish its tasks within a given time budget. On one side, the real-time planning system need expensive cost. On the other side, it is easily trapped in continuous re-planning and leads to low task success rate.

Recently, numerous path planning methods that consider uncertain human actions have been proposed to handle human-robot interactions. Chan’s research [30] presented a conflict-free speed alteration strategy for human safety. Although the superiority of this method has been shown by comparing it with two existing methods, this method plans one-dimensional moving paths, which is unsuitable for an autonomous system. Similar works, however, have made progress in the probabilistic prediction of a human’s motion [31, 32], and robust sequential trajectory planning for multirobot systems [33, 34]. However, fusing these techniques into a real-time planning system remains a challenge because of the difficulty in joint planning and prediction for multiple robots and humans. Bajcsy’s research [35] introduced a scalable framework for robot navigation that accounts for high-order system dynamics and maintains safety in the presence of external disturbances, other robots, and nondeterministic intentional agents. However, this framework decomposes multiagent path finding into a sequence of instances in which one replans paths at every time step for all agents [36, 37]. Such a framework maintains the human safety, but does not account for the time budget of robots, especially the success rate of tasks. Consequently, this framework is difficult to realize in real-world applications.

We summarize and make comparisons about the related path planning methods in Table 1. As described previously, the shortest path planning methods A^*^, D^*^, and RRT^*^ are not suitable for dynamic environments where human moves randomly. RL, FSA-MDP (Markov decision process) , and MCTS methods adopt learning schemes to obtain an optimal control policy, are efficient in static and dynamic environments. Moreover, those learning schemes are available for risk foresight that the future risk could be addressed in the learning phase through different ways such as stochastic simulations. Unfortunately, they suffer expensive computation cost and no optimality/safety is guaranteed. It is well known that real-time solutions are the safest approach to path planning which combines two key components: global path planning and real-time obstacle avoidance. The real-time method is widely applied in navigation. However, it lacks the risk foresight ability and cannot solve conflicts at the planning level. For warehouse scenarios, real-time obstacle obstacle avoidance can lead to low task success rate and the computation cost of online re-planning is also expensive. Above all, resolving conflicts at the planning level, rather than relying on real-time methods, is crucial for ensuring the safety of mobile robots working alongside humans while achieving a high task success rate.

**Table 1:** Comparison on different path planning methods.

Methods	Environment		Risk foresight
	Static	Dynamic	
A^*^	✓	—	—
D^*^	✓	—	—
RRT^*^	✓	—	—
RL	✓	✓	✓
FSA-MDP	✓	✓	✓
MCTS	✓	✓	✓
Real-time solution	✓	✓	—

It has been proved that syntactically co-safe linear temporal logic (scLTL), one fragment of linear temporal logic (LTL) [38], is efficient in task descriptions and control. Multiple tasks can be formulated using an scLTL formula [39–41], offering efficient methodologies for managing multi-task scenarios and seamlessly integrating with path planning methods, e.g., RL [42] in which the authors merge MDP with scLTL and propose an FSA-MDP algorithm to generate optimal paths against jamming attacks. Building upon this foundation, our primary aim is to generate safe and efficient plans that satisfy scLTL tasks for environments such as warehouses, factories, where humans and robots coexist or cooperate.

### 1.3 Our approach

**Fig 2:**
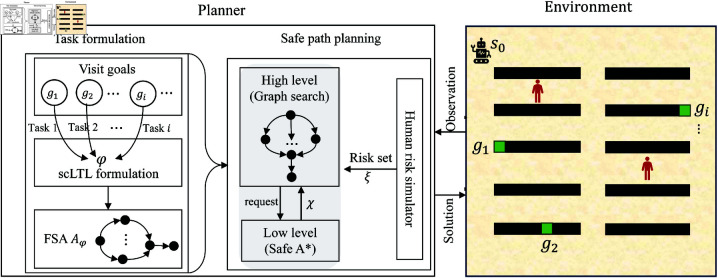
Architecture of the proposed safe planner.

To generate an optimal safe path that satisfy environments where humans and robots coexist or cooperate, e.g., warehouses, logistic centers, automatic factories, an efficient finite state automaton (FSA)-based risk search (FSARS) method considering environmental risks for mobile robot path planning in human-shared environments is proposed in this study. Compared with existing methods, in which only optimal path planning is considered, this method not only considers human safety but also efficiently accomplishes multiple tasks with lower collision risks. The contributions of this study are summarized as follows:

1. Our proposed method solves the conflicts at the planning level rather than aiming at real-time collision avoidance. The whole architecture of our proposed safe planner is shown in Fig 2.
2. A novel safe A^*^ method is proposed which enables a robot to find a local safe path instead of the shortest path with human risks in the low-level of FSARS.
3. In the high-level, a safety-first search structure is developed on the basis of FSA transitions to find global optimal safe paths while performing multiple tasks, ensuring the avoidance of collisions with humans moving stochastically.

The rest of this paper is organized as follows. Sect 2 briefly reviews the scLTL specifications and A^*^ method. Sect 3 introduces the problem formulation. Sect 4 describes the tasks control based on atomic proposition set. The proposed safe planner is presented in Sect 5, followed by the simulation results and discussions in Sect 6. Finally, we give a brief conclusion in Sect 7.

## 2 Preliminaries

### 2.1 scLTL

scLTL is one fragment of LTL. An scLTL formula commonly consists of a set of atomic propositions, Boolean operators, and temporal operators. Let *PrP* be the set of atomic propositions. An scLTL formula over the atomic propositions *PrP* is recursively defined as:

$$\varphi ::⊤\;|\;prp\;|\;\neg prp\;|\;\varphi_{1}\wedge \varphi_{2}\;|\;\varphi_{1}\vee \varphi_{2}\;|\;X\varphi \;|\;\varphi_{1}U\varphi_{2}.$$ (1)

Note that

- ⊥=¬⊤(False) is an scLTL formula,
- Fφ=⊤Uφ is an scLTL formula, and
- given an scLTL formula φ, it is written in a positive normal form where ¬ is only written in front of an atomic proposition *prp*.

¬ (negation), ∧ (conjunction), and ∨ (disconjuction) are Boolean operators. **X** (next), **F** (in the future), and **U** (until) are temporal operators. Combining the Boolean operators and temporal operators, complex and rich mission specifications can be described. An scLTL formula is interpreted by an infinite word $w\in (2^{PrP}{)}^{\omega }$ where w=w[0]w[1]w[2]…. Let w(i)=w[i]w[i+1]…, and semantics formula φ over word *w* is defined recursively in Eq (2) as follows:

$$w(i)⊧⊤,\;w(i)⊧c\Leftrightarrow c\in w,\;w(i)⊧\varphi_{1}\wedge \varphi_{2}\Leftrightarrow w(i)⊧\varphi_{1}\wedge w(i)⊧\varphi_{2},\;w(i)⊧\varphi_{1}\vee \varphi_{2}\Leftrightarrow w(i)⊧\varphi_{1}\vee w(i)⊧\varphi_{2},\;w(i)⊧X\varphi \Leftrightarrow w(i+1)⊧\varphi ,\;w(i)⊧\varphi_{1}U\varphi_{2}\Leftrightarrow \exists k\geq i,\forall j(i\leq j<k),w(k)⊧\varphi_{2}\wedge w(j)⊧\varphi_{1}.$$ (2)

For any scLTL formula φ, there exists an FSA ${𝒜}_{\varphi }=<Q,PrP,\delta ,q_{0},F>$ that accepts all infinite words satisfying the formula φ, where *Q* is the finite set of states, *PrP* is the set of atomic propositions, δ: $Q\;\times \;2^{PrP}\to Q$ is the state transition function, $q_{0}\in Q$ is the initial state, and F⊆Q is the set of the accepting states. Several off-the-shelf tools for the computation of ${𝒜}_{\varphi }$ have been developed [43][44].

### 2.2 Shortest path planning (SPP): A^*^ast

The SPP problem finding the shortest path to move an agent from a starting location to a goal location has already been studied well. A^*^ is an efficient algorithm for shortest path problems [45]. The following equation is the core function enabling A^*^ to perform best-first search.

f(v)=g(v)+h(v), (3)

where *g*(*v*) is the cost function that from a starting location to vertex *v*, and *h*(*v*) is the heuristic estimation from *v* to a goal location. A^*^ is one of the most efficient pathfinding methods for the SPP problem.

## 3 Problem formulation

We consider an agent working in an environment where humans move stochastically. The agent is assigned with *I*(*I*>1) tasks. Each task *i* defines one goal location *g*_*i*_ that the agent needs to visit. Any two tasks *i* and *j* (i≠j), are either *independent* or *sequential*. Task *i* is *independent* if task *i* can be executed without any consideration of task *j*. Otherwise, we say task *i* is a *sequential* task. For example, task *i* cannot be performed until task *j* is achieved.

The agent works in the environment shared with *K* humans. The humans move with a complete stochastic strategy (random policy) in the environment and cannot be controlled, which lead to the uncertainties for the agent visiting its goals. The agent may encounter a conflict with human *k* (k∈{1,…,K}) at any time-step *t* (we consider discrete time steps in this study).

Let

- *S* be the state set of the agent; $s_{t}=\{s_{t}\}\in S$ denote the state of the agent at time step *t*; *s*_0_ be the starting position; $s_{g}=\{g_{1},g_{2},\ldots ,g_{I}\}$ define the goal set corresponding to task set τ;
- *O*_*b*_ be the state set of obstacles which contains the positions that the agent cannot visit;
- 𝛤 be the task state set; ${𝛤}_{t}$ denote the task state at time step *t*; ${𝛤}_{0}=\{null_{1},null_{2},\ldots ,null_{I}\}$ define the initial task state, where *null*_*i*_ represents that task *i* is not achieved. We use $\tau_{i}$ to represent that task *i* is achieved, that is, ${𝛤}_{t}=\{null_{1},null_{2},\ldots ,\tau_{i},\ldots ,null_{I}\}$;
- *H* be the state set of humans; $h_{t}=\{h_{t}^{1},h_{t}^{2},\ldots ,h_{t}^{K}\}\in H$ denote the state of *K* humans at time step *t*;
- A={forward,backward,right,left,wait} define the action set of the agent and humans;
- $PrP=\cup_{i=1}^{I}PrP_{i}$ be the set of atomic propositions and $PrP_{i}=\{null_{i},\tau_{i}\}(1\leq i\leq I)$.

A *solution* is a path for the agent performing all tasks, written as $\pi =\{s_{0},s_{1},\ldots ,g_{i},g_{i+1},\ldots \}$, recording the trajectories from the start state *s*_0_ to a goal *g*_*i*_, then from goal *g*_*i*_ to goal *g*_*i* + 1_, etc. The agent performs *I* tasks within a time-step budget *T*. A conflict occurs when the agent and a human *k* occupy the same position or traverse the same edge, that is,

- a vertex conflict occurs when $s_{t}=h_{t}^{k}$, and
- an edge conflict occurs when $s_{t}=h_{t+1}^{k}\wedge s_{t+1}=h_{t}^{k}$.

A task is achieved only and only if an agent reaches its goal without collisions and within the time-step budget *T*. Otherwise, the task is failed. In a solution, if the agent collides with a human at time step *t* (no collision from time step 0 to *t*–1), only the goals visited within time step *t* are achieved, and those goals visited after time step *t* are failed.

In this study, we aim to find a safe solution to ensure that the agent visits all goals without collisions with humans. We pay more attention to the safety of the solution rather than the shortest path.

## 4 Task control based on FSA

Given *I independent* or *sequential* tasks, we formulate them in an scLTL formula φ based on the atomic proposition set *PrP*.

$$\varphi ={⋀}_{0<i,j\leq I,i\neq j}F\tau_{i}\wedge \;F\tau_{j}\wedge \;(\neg \tau_{j}U\tau_{i}),$$ (4)

where

- **F**$\tau_{i}$ and **F**$\tau_{j}$ represent that *independent* tasks *i* and *j* will be achieved in the future,
- $\neg \tau_{j}U\tau_{i}$ shows the relationship between *sequential* task *j* and task *i* that task *j* cannot be executed before task *i* is finished.

Note that $\neg \tau_{j}U\tau_{i}$ exists or not depending on the task setting. Afterward, an FSA ${𝒜}_{\varphi }=<Q,PrP,\delta ,q_{0},F>$ is built on the basis of φ for task control.

## 5 Proposed safe planner

### 5.1 Architecture

We propose a mobile robot safe planner and the architecture is illustrated in Fig 2. It mainly consists of two parts. One is the task formulation based on the built FSA ${𝒜}_{\varphi }=<Q,PrP,\delta ,q_{0},F>$, and the other one is safe path planning. The core part of the safe path planning is the FSARS algorithm. It calls a human risk simulator to generate a human risk set for risk handling. The proposed FSARS contains two layers. The low-level searches the optimal safe path considering human motion risks given a starting position and a goal. The high-level is used to find a global optimal safe path from the global view that considers all tasks. The details of the proposed safe path planning module is introduced as follows.

### 5.2 Human risk simulation

**Fig 3:**
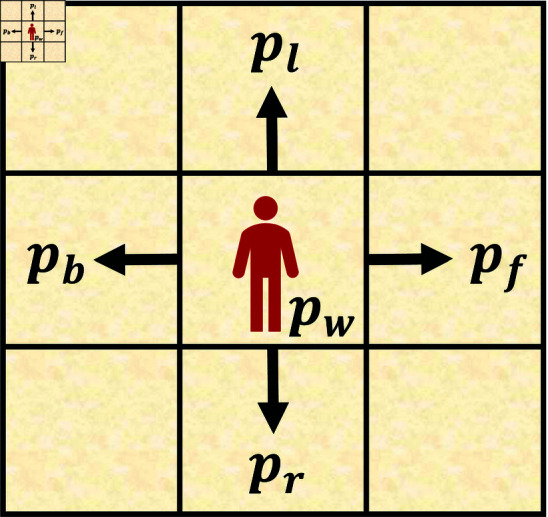
Stochastic model of a human. A human moves to his/her neighbors or take a “wait” action (*p*_*w*_) with a random policy.

The environment in this study is a 2D ($D_{x}\;\times \;D_{y}$) grid world. As described previously, we considered the most difficult scenario in which humans take complete stochastic strategies, that is, the humans move randomly. Fig 3 shows that a human takes an action *a* (a∈A) with a probability *p*_*a*_, and $p_{a}\in \{p_{f},p_{b},p_{r},p_{l},p_{w}\}$ is the probability set with respect to action set *A*. *p*_*a*_ is unknown as the human moves with a fully random policy.

In this study, we perform random simulations to calculate the human risks, which include handles complex scenarios but are simple in terms of calculation. For a human *k*, let $h_{t_{0}}^{k}$ be the observed state at time step *t*_0_, where $h_{t_{0}}^{k}=v$ means that a vertex v=(x,y) (vertex index x,y∈ℕ) is occupied. We run stochastic simulations from the observed state $h_{t_{0}}^{k}$ to predict a human’s paths and calculate the conditional probability of a vertex *v* occupied by the human *k* at time step $t\;(t_{0}<t\leq t_{0}\;+\;T)$. It is naively calculated as the ratio of the visited times of the vertex *v* at time step *t* to the simulation times, as shown as follows:

$$p_{k,t}(v|h_{t_{0}}^{k})=\frac{\text{visited times of }v}{\text{simulation times}}.$$ (5)

**Fig 4:**
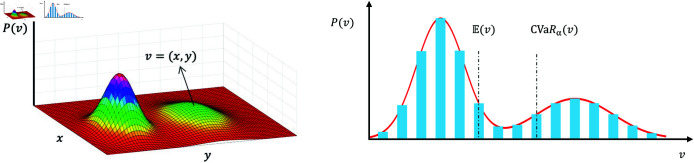
Probability distribution of humans at time step t. (a) Example of the probability distribution. (b) PMF over the vertices of a 2D grid map.

Fig 4 shows the probability mass function (PMF). Apparently, the probability distribution of human risks is complex and includes multiple peaks. Many studies have been conducted on risk calculation, such as expectation method [46] and conditional value-at-risk (CVaR) [47][48]. However, the expectation method cannot address the case with multiple peaks and CVaR is difficult to calculate. In this study, we simulate *K* humans to calculate the risk value of vertex *v* that occupied by humans, defined by

$$R(v,t)=\sum_{k=1}^{K}p_{k,t}(v|h_{t_{0}}^{k}).$$ (6)

**Fig 5:**
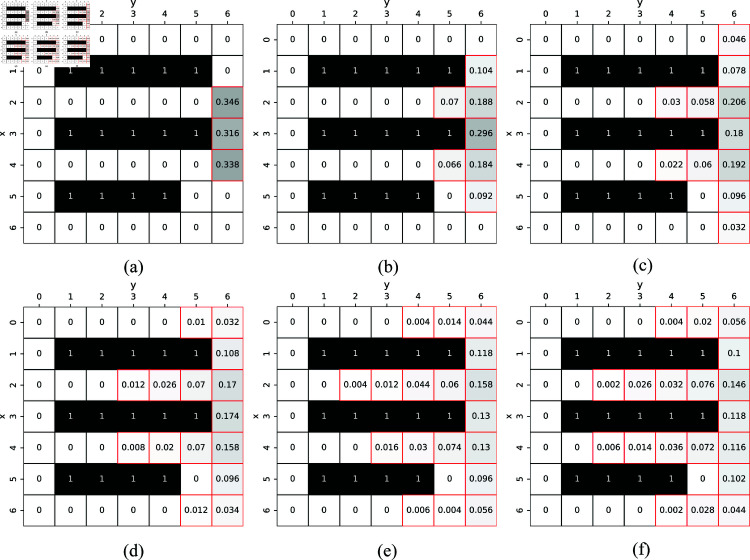
Human risk map $\xi =\{\xi_{1},\xi_{2},\ldots ,\xi_{6}\}$. The human is observed in position (3,6) at time step *t* = 0. Black squares denote obstacles. We simulate 2000 times to calculate the human risk. The numbers marked by red squares are risks. For example, in subfigure (a), R((2,6),1)=0.346 represents that the risk associated with the vertex v=(2,6) at time step *t* = 1 is 0.346. (b) *t* = 2, (c) *t* = 3, (d) *t* = 4, (e) *t* = 5, and (f) *t* = 6.

Finally, we obtain human risk set $\xi =\{\xi_{t_{0}+1},\xi_{t_{0}+2},\ldots ,\xi_{t_{0}\;+\;T}\}$ from time step *t*_0_ to $t_{0}\;+\;T$. We can easily determine the risk of each vertex *v* at any time step *t* from $\xi_{t}$. Fig 5 shows an example: a scenario of one human in a 7×7 grid world. The starting position of the human $h_{0}^{1}$ is (3,6). We show the risk map from time step *t* = 0 to *t* = 6. The simulated human risk sets $\xi_{1},\xi_{2},\ldots ,\xi_{6}$ are shown in Figs 5a, 5b, …, and 5f, respectively. Thus far, the human risk distribution with multiple peaks is handled in the way above.

### 5.3 Low-level: Safe A^*^ast pathfinding under human risks

A^*^ is an efficient algorithm for SSP [45]. However, it cannot handle a map with dynamic changes. To generate a safe path, we develop a safe A^*^ method that performs best-first search, as well as A^*^. The core idea is to take human risks into consideration. The evaluation function *f*(*v*) is redefined by

f(v)=g(v)+h(v)+z(v), (7)

where *g*(*v*) is the cost function that from the starting location to location *v* (time step is *t*), *h*(*v*) is the heuristic estimation from *v* to the goal location, and *z*(*v*) is the additional risk cost from humans. The risk cost is not only determined by the location *v* but also affected by the future risk. *z*(*v*) is calculated by

$$z(v)=V_{r}(v)+E_{r}(v)+F_{r}(v),$$ (8)

where $V_{r}(v)$ is the vertex conflict risk at location *v*, $E_{r}(v)$ is the edge conflict risk from the current location *v* to the next location $v^{\prime }$ whose time step is t+1, and $F_{r}(v)$ is the future risk. $V_{r}(v)$ and $E_{r}(v)$ are calculated using Eqs 9 and (10), respectively

$$V_{r}(v)=R(v,t),$$ (9)

$$E_{r}(v)=R(v,t+1)*R(v^{\prime },t).$$ (10)

Given a deterministic path, the future risk $F_{r}(v)$, theoretically, is theoretically calculated by

$$F_{r}(v)=V_{r}(v^{\prime })+E_{r}(v^{\prime })+V_{r}(v^{\prime \prime })+E_{r}(v^{\prime \prime })+\ldots ,$$ (11)

where $v^{\prime \prime }$ is the location at time step t+2, and $F_{r}(v)$ is the accumulated risk from the current location until arriving at the goal. However, we have no knowledge about the vertices that the agent will visits in the future. In other words, we cannot use Eq 11 to calculate the future risk. Instead, we take an approximation method to measure the future risk by

$$F_{r}(v)=\sum_{\bar{v}\in neighbours}\left(V_{r}(\bar{v})+E_{r}(\bar{v})\right).$$ (12)

As shown in Eq 7, *g*(*v*) and *h*(*v*) are the distances, and *z*(*v*) is the risk value that is usually much smaller than *g*(*v*) and *h*(*v*). We need balance parameters, such that

$$f(v)=g(v)+h(v)+\frac{1}{\gamma }z(v),$$ (13)

where γ is a scale parameter to balance the future risk cost and distances.

### 5.4 High-level: Searching an FSA-based graph

In the high-level, FSARS searches an FSA-based graph to plan a global optimal safe path for the agent. The FSA ${𝒜}_{\varphi }=<Q,PrP,\delta ,q_{0},F>$ can easily be obtained. For more details, please see our previous work [42]. In the high-level, each node *n* contains,

- *n*.*q*, one state of the FSA ${𝒜}_{\varphi }$,
- *n*.*s*, a goal state of the robot,
- *n*.*path*, the path from its parent to the current node,
- *n*.*value*, the value of current node, used to evaluate the safety of the node,
- *n*.*p*, parent node of *n*.

Each edge represents a path χ from one node to its successor generated by the low-level.

**Fig 6:**
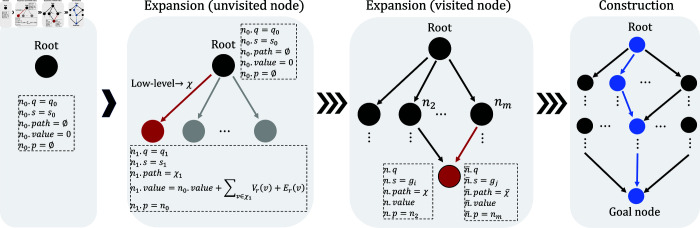
High-level search process of the proposed FSARS.

In FSARS, the high-level is a graph-based search method. Its search process is illustrated in Fig 6. The search graph starts from root node *n*_0_ as the selection phase shown in Fig 6 (left). For root node *n*_0_,

- $n_{0}.q=q_{0}$ (*q*_0_ is the initial state of FSA ${𝒜}_{\varphi }$),
- $n_{0}.s=s_{0}$ is the initial state of the robot,
- *n*_0_.*path* is empty in the root node,
- $n_{0}.value$ is 0, and
- $n_{0}.p=\emptyset$ as it has no parent node.

***Selection*** This phase determines which node to expand. The high-level performs safety-first search, in which FSARS always selects a node with minimum the value; the lower *n*.*value* is, the safer the node is. For each exploration, we choose the node from the root using

$$selected\_node=arg\min_{n^{\prime }\in successors}(n^{\prime }.value).$$ (14)

If a node is chosen to expand for the first time, that is, a leaf node, none of its *successors* have been visited, one *successor* will be chosen with a random policy. If all *successors* of a node have been visited, then the node is fully expanded. Eventually, a leaf node or a child node not fully expanded is selected for expansion. Note that we evaluate each node by its value *n*.*value*, and take a safety-first policy. The proposed algorithm does not have to consider the balance of exploration and exploitation as UCB/UCT performs in Bandit problem.

***Expansion*** Each node *n* is expanded on the basis of the transitions of FSA ${𝒜}_{\varphi }$, from which the *successors* are obtained. We choose a $successor\;n^{\prime }$ with minimum $n^{\prime }.value$ to expand the node *n*, which is called the parent node of $n^{\prime }$.

For a $successor\;n^{\prime }$,

- $n^{\prime }.q=q^{\prime }$ where $q^{\prime }$ is obtained from ${𝒜}_{\varphi }$ (for transition details see δ(·) in [42]),
- $n^{\prime }.s=g_{i}$,
- $n^{\prime }.path=\chi$ which is planed by the low-level,
- $n^{\prime }.value$ contains two parts. One part is inherited from its parent node, and the other part is determined by $n^{\prime }.path$. Eq 15 shows how $n^{\prime }.value$ is calculated,
- $n^{\prime }.p=n$.

$$n^{\prime }.value=n^{\prime }.p.value+\sum_{v\in n^{\prime }.path}\left(V_{r}(v)+E_{r}(v)\right).$$ (15)

The expansion (unvisited node) in Fig 6 shows an expansion example of expanding root node *n*_0_ to node *n*_1_. This is how an unvisited *successor* expanded.

Note that one node in FSARS can be visited many times. As shown in the expansion (visited node) of Fig 6, the red node is already visited before the expansion from node *n*_*m*_. The existed expansion is from node *n*_2_ where

- *n*.*q* = *q*,
- *n*.*s* = *g*_*i*_,
- n.path=χ,
- n.value← Eq 15,
- *n*.*p* = *n*_2_.

The red edge and node shows the new expansion where

- $\bar{n}.q=q$ ,
- $\bar{n}.s=g_{j}$,
- $\bar{n}.path=\bar{\chi }$,
- $\bar{n}.value\leftarrow$ Eq 15 ,
- $\bar{n}.p=n_{m}$.

As the expansion (visited node) of Fig 6 shows, one expansion is from node *n*_2_, the other one is from *n*_*m*_, the two expansions are quite different except $n.q=n^{\prime }.q$, in details,

- the goals are different, $n.s\neq \bar{n}.s$, although they share the same *q* state,
- the paths are different, $\chi \neq \bar{\chi }$, as the goals in two expansions are different,
- the most important is that the values may be different.

In FSARS, the lower n.value is, the safer the node is. If $\bar{n}.value<n.value$, that is the new expansion is safer than previous one, we replace the previous expansion information with new expansion where

- *n*.*q* = *q*,
- $n.s\leftarrow \bar{n}.s=g_{j}$,
- $n.path\leftarrow \bar{n}.path=\bar{\chi }$,
- $n.value\leftarrow \bar{n}.value$,
- $n.p\leftarrow \bar{n}.p=n_{m}$.

Otherwise, we keep the previous node information.

***Construction*** The proposed FSARS performs safety-first search. The search process ends at the goal node eventually. After the search process is finished, FSARS traverses the graph from the goal node to the root and construct the path. As shown in Fig 6 (right), the blue nodes and edges are selected to construct the path of the agent. The whole process of FSARS is presented in Algorithm 1.

**Algorithm 1.** High-level algorithm.

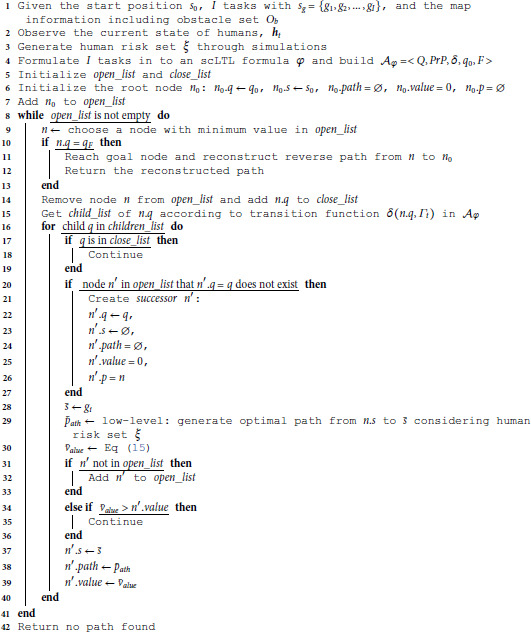



## 6 Simulations and discussions

To verify the proposed methods, we conduct different scenario simulations and compare the simulation results of various methods. All simulations are conducted on a 13-inch Macbook Pro with M2 chip and 16 GB memory. For all methods in verification, we use the same reward design, and the reward definitions are as follows:

- goal reward *r*_*g*_ = 1.0,
- step penalty *r*_*s*_ = −0.1,
- conflict penalty *r*_*c*_ = −0.5.

Once the agent achieves one task, it obtains a goal reward *r*_*g*_. In the meanwhile, the agent receives a step penalty *r*_*s*_ for each step, also called step cost. Because the environment is shared with humans, the agent is penalized with *r*_*c*_ when it collides with a human.

We evaluate the performance of the proposed methods from different aspects, such as conflict number, conflict distribution, task success rate, reward, and runtime. In each scenario, we simulate 100 times and calculate the average value for evaluation. We also conducted comparison simulations using classic A^*^, RL/FSA-MDP, and MCTS methods. RL is widely applied in pathfinding for static and dynamic environments, FSA-MDP is efficient in multitask pathfinding, and MCTS is famous for handling dynamics. Note that RL and FSA-MDP are similar in the case of single task.

### 6.1 Scenario 1: Verification of the low-level safe A^*^ast

**Fig 7:**
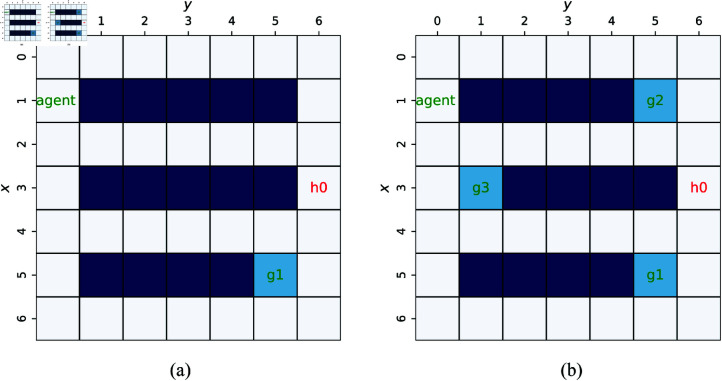
Simulation environmental setting with a 7 × 7 grid map, and human number K=1. (a) Task number *I* = 1. (b) Task number *I* = 3.

In scenario 1, the simulation is designed to verify the proposed safe A^*^, the low-level of FSARS. Only one task is assigned to the agent, and the high-level does not work. The simulation environment, as shown in Fig 7a, is a 7×7 grid world. The agent starts from *s*_0_ = (1,1), and *g*_1_ = (5,5) is its goal position. A human moves stochastically from position $h_{0}^{1}=(3,6)$. The time budget *T* = 20.

The parameter of Eq 13 γ=0.01 which is obtained from experience. We firstly investigate the effect of γ and show its performances on conflict number and task success rate where γ varies from 1 to 0.001 in the environment as Fig 7a. The important points of γ are illustrated in Table 2. The conflict number decreases when γ becomes smaller until γ=0.01. In the meanwhile, the task success rate increases. γ=0.01 is a turning point. The conflict number stays around 0.05 even γ becomes smaller. However, the task success rate decreases greatly after γ=0.01. This is because the safe A^*^ pays too much attention on human risks. Once the risk cost is scaled too much, safe A^*^ takes expensive step cost to avoid the risks and the agent

**Table 2:** Investigation on performances of parameter γ.

γ	Conflict number	Task success rate
—	0.28 ± 0.53	0.76 ± 0.43
1.0	0.22 ± 0.52	0.83 ± 0.38
0.5	0.21 ± 0.49	0.83 ± 0.38
0.1	0.19 ± 0.47	0.84 ± 0.37
0.05	0.09 ± 0.34	0.92 ± 0.27
0.01	0.05 ± 0.22	0.95 ± 0.22
0.005	0.05 ± 0.35	0.00 ± 0.00
0.001	0.05 ± 0.35	0.00 ± 0.00

**Note:** The additional risk cost from humans, *z*(*v*), is not considered when γ=−.

cannot accomplish its task within the time budget. From Table 2, we can see that, γ=0.01, has good performances in balancing conflict and task success rate. In the following simulations, we set γ=0.01.

**Fig 8:**
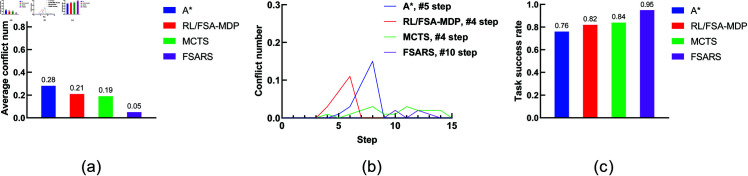
Results of scenario 1 with the environmental setting of 1 task, 1 human, and a 7 × 7 grid map. (a) Average conflict number. (b) Conflict distribution. (c) Average task success rate.

The simulation results are summarized in Table 3. Fig 8 shows the comparison. We analyze the performance as follows.

**Table 3:** Results of average conflict number, average task success rate, average reward, and average runtimeeak (in seconds) using different methods with the environmental setting of 1 task, 1 human, and a 7×7 grid map.

Methods	Conflict number	Task success rate	Reward	Runtime
A^*^	0.28 ± 0.53	0.76 ± 0.43	–0.04 ± 0.27	<0.01
RL/FSA-MDP	0.21 ± 0.48	0.82 ± 0.38	–0.11 ± 0.24	1.17 ± 0.02
MCTS	0.19 ± 0.46	0.84 ± 0.37	–0.10 ± 0.23	12.6 ± 4.68
FSARS	0.05 ± 0.22	0.95 ± 0.22	–0.19 ± 0.16	0.02 ± 0.01

**(1) Conflict number.** Fig 8a shows the average conflict number in 100 rounds of simulations. A^*^ method has the highest conflict numbers (0.28) compared with the other methods. A^*^ generates the shortest path in static environments. It does not consider any information of the humans’ movement. Both RL/FSA-MDP and MCTS consider the humans’ movements, and they perform better than A^*^. The proposed FSARS method has the least average conflict number, 0.05, which is much less than the ones of the other three methods. The reason is that safe A^*^ takes human risk set ξ in to consideration. Apparently, the proposed safe A^*^ method outperforms the other methods in finding a safe path, which greatly reduces the number of conflict with humans.

**(2) Conflict distribution.** Fig 8b shows the conflict distribution that records the time step of the conflicts that occur. In A^*^, the first conflict occurs at time step *t* = 5. In RL/FSA-MDP and MCTS, the first conflict occurs at time step *t* = 4. The first conflict occurs almost at the same time step in the above three methods. The first conflict that occurs in the proposed method is much farther behind than those in the other methods, which is at time step *t* = 10. This results shows that the proposed safe A^*^ not only reduces the conflict number but also delays the time at which a conflict occurs. Actually, the word “delay” is not accurate. Safe A^*^ avoids the dangerous positions within time step *t* = 10 so that no conflict occurs before *t* = 10. In other words, safe A^*^ can generate a safe path within a time-horizon window in which no conflict occurs.

**(3) Task success rate.** In this study, task success rate is another important evaluation indicator. The agent should accomplish its tasks within a given budget *T*. From Fig 8c, the proposed method, with the highest success rate of 0.95, outperforms the other methods.

**(4) Reward and runtime.** The average reward and runtime are shown in Table 3. Many studies have regarded reward and runtime as important evaluation indicators. However, we pay more attention to conflict number, conflict distribution, and task success rate because our main purpose is the safety of the generated path. From Table 3, we can see that A^*^ has the highest reward because it plans the shortest path without consideration of conflicts with humans. The proposed method obtains the lowest reward but with least conflicts. The proposed safe A^*^ needs to avoid the positions with high conflict risk. Unavoidably, the length of the generated path by safe A^*^ is longer than those by the other methods. As for the runtime, A^*^ is the fastest one with the runtime <0.01 s. Actually, comparing these four methods is unfair because they use different schemes. The proposed FSARS takes 0.02 s to generate a safe path. Given that FSARS needs to handle the human risks, this result is good, despite FSARS is not as fast as A^*^.

**Fig 9:**
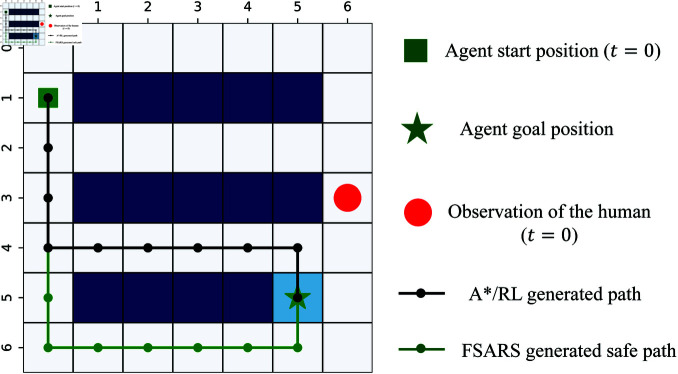
An example of generated safe path.

Figure 9 shows an example of the generated paths. The red circle is the observed position of a human at *t* = 0. The black line shows the planed path by the A^*^/RL methods which is the shortest path. The green line is one safe path generated by FSARS. It is not the shortest path; however, it is more safe than the shortest path for the agent. Selecting the green path, the agent faces a lower conflict risk with the human under the observation.

The simulation results demonstrate that the proposed low-level safe A^*^ can generate a safe path with minimal conflict and high task success rate in a scenario including a starting position and a goal. Moreover, the safe A^*^ can generate a conflict-free path within a time-horizon window.

### 6.2 Scenario 2: Verification of the proposed FSARS

**Fig 10:**
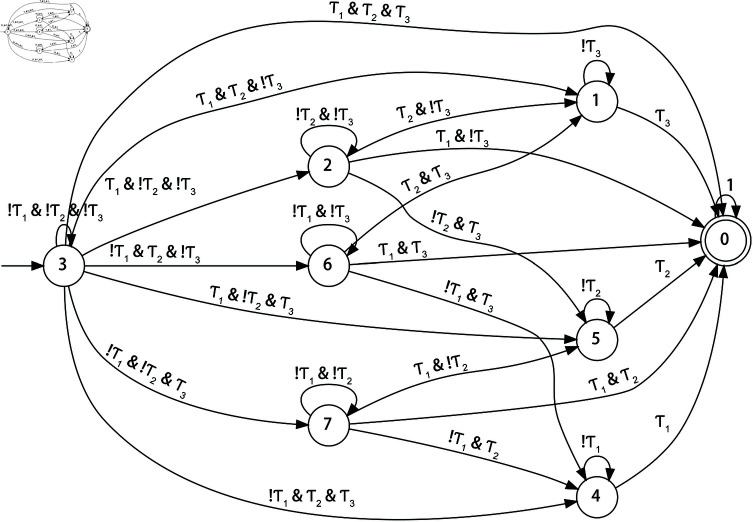
FSA ${𝒜}_{\varphi }$ state transitions.

Simulations of scenario 1 have verified the efficiency of the low-level safe A^*^ method. Scenario 2 is designed to verify both the low-level and the high-level of FSARS and the performances with maps of different sizes. We conducted three simulations in scenario 2:

- Simulation environment 1: one agent, one human, three tasks, a 7×7 grid map (Fig 7b), budget *T* = 50, and the built FSA ${𝒜}_{\varphi }$ is shown in Fig 10,
- Simulation environment 2: one agent, one human, three tasks, a 10×10 grid map, budget *T* = 50,
- Simulation environment 3: one agent, one human, three tasks, a 20×20 grid map, budget *T* = 100.

The state transitions of the built FSA ${𝒜}_{\varphi }$ (*I* = 3) are shown in Fig 10, based on which the proposed FSARS performs safety-first search.

**Fig 11:**
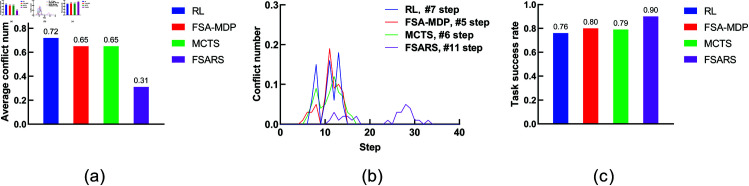
Results of the environmental setting of 3 tasks, 1 human, and a 7 × 7 grid map. (a) Average conflict number. (b) Conflict distribution. (c) Average task success rate.

**Fig 12:**
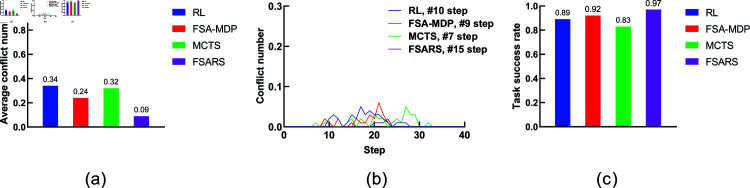
Results of the environmental setting of 3 tasks, 1 human, and a 10 × 10 grid map. (a) Average conflict number. (b) Conflict distribution. (c) Average task success rate.

**Fig 13:**
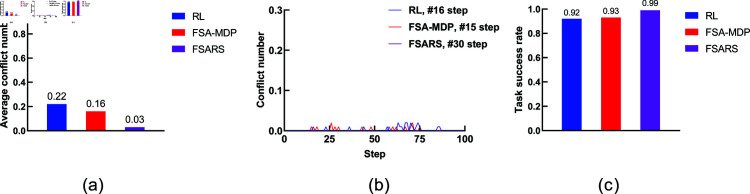
Results of the environmental setting of 3 tasks, 1 human, and a 20 × 20 grid map. (a) Average conflict number. (b) Conflict distribution. (c) Average task success rate.

The simulation results are summarized in Tables 4, 5, and 6, and Figs 11, 12, and 13. MCTS consumes excessive time in simulation environment 3, so we do not show the results in Table 6 and Fig 13.

**Table 4:** Average results under the environmental setting of 3 tasks, 1 human, and a 7×7 grid map.

Methods	Conflict number	Task success rate	Reward	Runtime
RL	0.72 ± 0.71	0.76 ± 0.24	1.14 ± 0.35	3.13 ± 0.04
FSA-MDP	0.65 ± 0.79	0.80 ± 0.23	1.18 ± 0.40	2.65 ± 0.04
MCTS	0.65 ± 0.95	0.79 ± 0.25	1.09 ± 0.52	426.71 ± 146.33
FSARS	0.31 ± 0.60	0.90 ± 0.19	–0.27 ± 0.37	0.11 ± 0.01

**Table 5:** Average results under the environmental setting of 3 tasks, 1 human, and a 10×10 grid map.

Methods	Conflict number	Task success rate	Reward	Runtime
RL	0.34 ± 0.62	0.89 ± 0.20	0.43 ± 0.31	6.5 ± 0.13
FSA-MDP	0.24 ± 0.53	0.92 ± 0.18	0.48 ± 0.27	5.39 ± 0.10
MCTS	0.32 ± 0.65	0.83 ± 0.27	–0.39 ± 0.49	1450.81 ± 504.61
FSARS	0.09 ± 0.29	0.97 ± 0.11	0.12 ± 0.20	0.06 ± 0.01

**Table 6:** Average results under the environmental setting of 3 tasks, 1 human, and a 20×20 grid map.

Methods	Conflict number	Task success rate	Reward	Runtime
RL	0.22 ± 1.02	0.92 ± 0.20	–4.49 ± 1.82	36.52 ± 0.43
FSA-MDP	0.16 ± 1.21	0.93 ± 0.20	–2.94 ± 2.01	33.15 ± 0.33
FSARS	0.03 ± 0.17	0.99 ± 0.08	–2.89 ± 0.41	0.13 ± 0.02

**(1) Conflict number.** Figs 11a, 12a, and 13a show the average conflict number of each method in the three simulation environments. Apparently, the proposed FSARS method has the best performance with least conflict numbers in all simulations. In simulation environment 1, the conflict numbers are reduced by more than 50% using our proposed FSARS method compared with those using RL, FSA-MDP, and MCTS methods. When the map changes from crowded to sparse (that is, the grid map changes from 7×7 to 20×20), the average conflict numbers are all reduced, and the proposed FSARS performs the best. The conflict numbers are reduced by more than 60% and 80% in simulation environments 2 and 3, respectively. This results is a great improvement in reducing conflict numbers in human-shared environments compared with those when using traditional methods such as RL, FSA-MDP, and MCTS. The simulations prove that our proposed FSARS can improve the safety of the generated path by reducing conflicts with humans.

**(2) Conflict distribution.** Figs 11b, 12b, and 13b show the conflict distributions of the three simulations in scenario 2. The results are almost the same as those in scenario 1, in which the occurrence time of the first conflict in FSARS is the latest one. Our proposed FSARS still works well when the task number increases from *I* = 1 to *I* = 3 and the map size increases from 7×7 to 20×20. Here, we take Fig 11b as an example. Generating a complete conflict-free path for an agent assigned with multiple tasks in a crowded human-shared environment is difficult. To the best of our knowledge, there is no one algorithm can provide the guarantee of generating a conflict-free path in an environment with stochastic human movements. Our proposed FSARS does not aim to generate a conflict-free path for executing multiple tasks while planning only one time. We aim to reduce the number of conflicts with humans and try to generate a conflict-free path within a time-horizon window. Fig 11b demonstrates that the proposed method does not encounter a conflict before step *t* = 11, in comparison with RL, FSA-MDP, and MCTS. Figs 12b and 13b show that conflict does not occur before time step *t* = 15 and *t* = 30 in FSARS generated paths under the simulation environments 2 and 3, respectively. The simulation results prove that our proposed FSARS outperforms the traditional methods in generating a safer path. The more important aspect is that the FSARS can generate a conflict-free path within a time-horizon window.

**(3) Task success rate.** Figs 11c, 12c, and 13c show that the proposed FSARS method achieves the highest success rate in all three simulation environments and prove the efficiency of the proposed FSARS in achieving multiple tasks.

**Fig 14:**
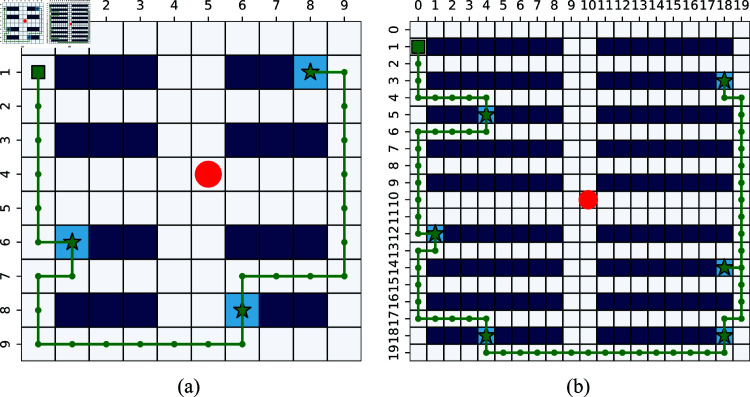
Generated paths of FSARS: (a) 10×10 map, 3 tasks and 1 human, (b) 20×20 map, 6 tasks and 1 human. Red circles are observed positions of humans. Green square is the start position and the stars are goals of the agent.

**(4) Reward and runtime.** The average reward and runtime are shown in Tables 4, 5, and 6. From Table 4, we can see that FSARS still obtains the smallest reward in a small-size map, and the results are the same as those in scenario 1. When the map size becomes 10×10, the reward of FSARS is higher than that of the MCTS method but is lower than those of the RL and FSA-MDP methods. The FSARS outperforms RL and FSA-MDP when we enlarge the map size to 20×20. The simulation results show a trend that the proposed FSARS performs better in a large map from the view of reward. As for the runtime, the FSARS is the fastest one. FSA-MDP is faster than RL, and MCTS is the slowest because it needs huge simulations. Fig 14a shows an example of FSARS generated path in a 10×10 grid map with 3 tasks and 1 human. Fig 14b shows an example of FSARS generated path in a 20×20 grid map with 6 tasks and 1 human. The generated paths show that the agent visits its goals while avoiding dangerous areas.

The simulation results in scenario 2 demonstrate that the proposed FSARS method works well in small- and large-size maps with less conflicts and a higher task success rate than the other methods. The efficiency of FSARS in generating a safe path is further proved.

### 6.3 Scenario 3: Verification of FSARS by increasing human number

**Fig 15:**
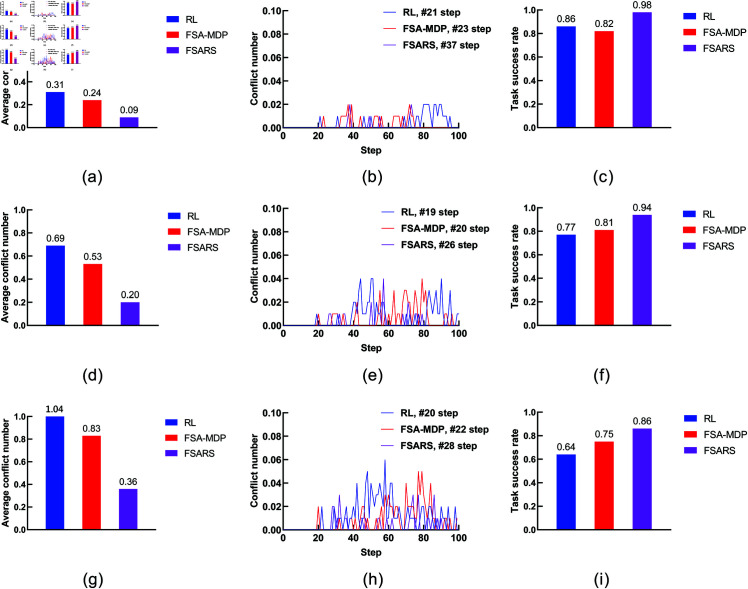
Results of the environmental setting of 6 tasks, 1~3 human, and a 20 × 20 grid map. (a), (d) and (g) are the average conflict number with K=1,2,3, respectively. (b), (e) and (h) are the conflict distribution with K=1,2,3, respectively. (c), (f) and (i) are the average task success rate with K=1,2,3, respectively.

In this scenario, we investigate the performances of FSARS with increasing human numbers. The environment is a 20×20 grid map and the agent is assigned with task number *I* = 6. We increase the human number *K* from 1 to 3. The simulation results are shown in Table 7 and Fig 15.

**Table 7:** Average results under the environmental setting of 6 tasks, 1 ~ 3 human, and a 20×20 grid map.

Human number	Methods	Conflict number	Task success rate	Reward	Runtime
*K* = 1	RL	0.31 ± 0.95	0.86 ± 0.15	–4.52 ± 1.12	69.87 ± 0.83
	FSA-MDP	0.24 ± 0.85	0.82 ± 0.29	–3.47 ± 2.26	52.65 ± 0.61
	FSARS	0.09 ± 0.35	0.98 ± 0.11	–2.19 ± 0.56	0.68 ± 0.10
*K* = 2	RL	0.69 ± 1.50	0.77 ± 0.22	–4.79 ± 1.42	121.62 ± 1.94
	FSA-MDP	0.53 ± 1.27	0.81 ± 0.26	–3.51 ± 2.27	92.01 ± 1.46
	FSARS	0.20 ± 0.55	0.94 ± 0.14	–2.38 ± 0.84	1.44 ± 0.11
*K* = 3	RL	1.04 ± 1.56	0.64 ± 0.23	–5.40 ± 1.65	173.90 ± 3.05
	FSA-MDP	0.83 ± 1.92	0.75 ± 0.27	–4.10 ± 2.63	127.95 ± 2.12
	FSARS	0.36 ± 0.64	0.86 ± 0.21	–3.24 ± 0.90	2.92 ± 0.16

**(1) Conflict number.** As Table 7 and Fig 15 show, for all methods, the average conflict number increases with increasing human number *K*. When K=1,2,3, the average conflicts of FSARS are 0.09, 0.2, and 0.36, respectively; the average conflicts of RL are 0.31, 0.69, and 1.04, respectively; the average conflicts of FSA-MDP are 0.24, 0.53, and 0.83, respectively. As shown in Figs 15a, 15d, and 15g, the average conflict number is reduced 71.0%, 71.0%, and 65.4% by FSARS, comparing with the one of RL, with respect to K=1,2,3. Apparently, the proposed FSARS performs the best and has the least number of conflicts in each case of different numbers of humans.

**(2) Conflict distribution.** Figs 15b, 15e, and 15h show the conflict distributions of the simulations with K=1,2,3. The results are consistent with the results of previous simulations that the proposed FSARS performs the best. When *K* = 1, no conflict occurs before step *t* = 37 in the path generated by FSARS whereas *t* = 21 in RL and *t* = 23 in FSA-MDP. When *K* = 2, no conflict occurs before step *t* = 26 in the path generated by FSARS whereas *t* = 19 in RL and *t* = 20 in FSA-MDP. When *K* = 3, no conflict occurs before step *t* = 28 in the path generated by FSARS whereas *t* = 20 in RL and *t* = 22 in FSA-MDP. Compared with the case *K* = 1, when *K* = 2, the time step of conflict occurrence is much earlier for all methods. Conflicts occur earlier and the conflict number increases when the number of human in the environment increases. When *K* = 3, the first conflict in FSARS occurs at time step *t* = 28, slightly later than when *K* = 2. The other two methods are similar. For the RL method, the first conflict occurs almost at the same time step in the cases of *K* = 2 and *K* = 3. Based on our analysis, the starting position of the third human added leads to this result.

**(3) Task success rate.** Figs 15c, 15f, and 15i show that the success rate decreases when the human number *K* increases for the three methods. However, the proposed FSARS method still achieves the highest success rate. Compared with RL, the task success rate of FSARS improves 34.4%, as shown in Fig 15i.

**(4) Reward and runtime.** The average reward and runtime are shown in Table 7. The reward decreases as the human number *K* increases. Compared with the two methods, FSARS obtains the highest reward for each case of *K*. As for the runtime, the computational time increases as *K* increases. The reason is that the uncertainties increases when the human number increases. FSARS runs much faster and the computational time is less than 3 s when *K* = 3, whereas the other two methods need more than 2 min.

The human number *K* has more influence on the runtime than the map size for the proposed FSARS method. As is known, the size of the search space is $D_{x}\times D_{y}\times 2^{I}$. The map size and task number affect the runtime by enlarging the search space. The human number affects the runtime by increasing the search difficulty. With more humans in the environment, the uncertainties become stronger, and it is more difficult to search for a safe path. From Tables 5 and 6, the computational time of the 20 grid map is 0.13 s, which is about the 2 times of the computational time of the 10 grid map while the map size becomes 4 times of that of the 10 grid map. From Table 7, we can see that the runtime of FSARS with *K* = 2 is about 2 times of that with *K* = 1, and the runtime of that with *K* = 3 is about 2 times of that with *K* = 2. Thus, the runtime becomes 2 times longer when one human is added with more.

**Fig 16:**
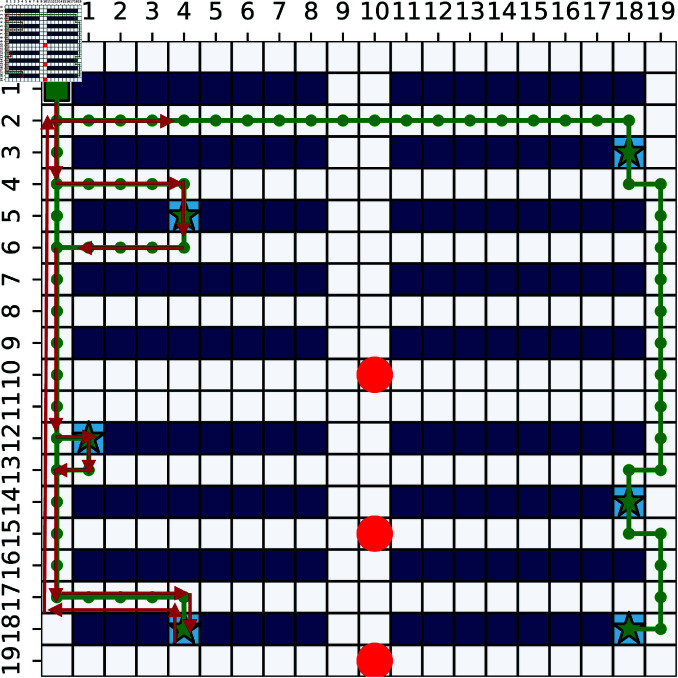
Generated path with 20×20 map, 6 tasks and 3 humans. Red arrows show the details of the overlapped part of the path.Red circles are observed positions of humans. Green square is the start position and the stars are goals of the agent.

Figure 16 shows one example of FSARS generated safe path. Part of the generated path is overlapped and the red arrows show the details. As shown in Fig 16, the generated path guides the agent to its goals without crossing the middle areas where are with high conflict risk with humans.

Table 7 mainly shows the investigation on increasing human number. As the number of humans increases, the average conflict number rises, while the average task success rate and reward decrease, and the runtime extends. The proposed FSARS is more robust than RL and FSA-MDP methods in solving conflicts and achieving high task success rate.

**Fig 17:**
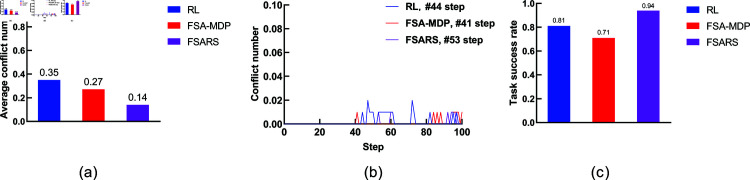
Results of the environmental setting of 6 tasks, 3 human, and a 40 × 40 map size. (a) is the average conflict number. (b) is the conflict distribution. (c) is the average task success rate.

**Fig 18:**
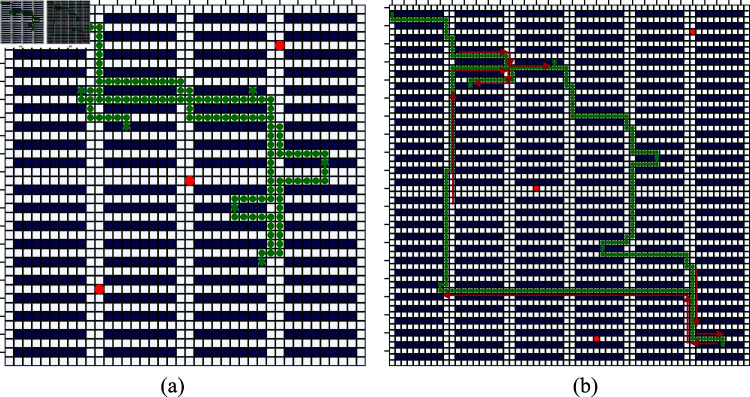
Generated paths of FSARS: (a) 40×40 map, 6 tasks and 3 humans, (b) 60×60 map, 6 tasks and 3 humans. Red arrows show the move directions.

We then verified our method with large maps, 40×40 and 60×60. The results are shown in Fig 17 and Table 8. The performance of FSARS is better than the other two methods in both conflict number and task success rate in the map of 40×40. For map of 60×60, the search space is $60\;\times \;60\;\times \;2^{6}=230,400$ with is large. As Table 8 shows, FSARS still works well and returns good results with low conflict number 0.09 and high task success rate 0.98. The average runtime increases to only 10.27 s. In contrast, the RL and FSA-MDP methods fail to learn an optimal path within a runtime budget 3600 s. Fig 18 shows examples of generated paths for map size of 40×40 and 60×60. The results show the effectiveness of our method in addressing large maps.

**Table 8:** Average results under the environmental setting of 6 tasks, 3 humans, and large map size.

Map size	Methods	Conflict number	Task success rate	Reward	Runtime
40×40	RL	0.35 ± 1.05	0.81 ± 0.32	–10.88 ± 2.77	660.84 ± 31.78
	FSA-MDP	0.27 ± 0.86	0.71 ± 0.39	–10.15 ± 6.03	463.57 ± 20.22
	FSARS	0.14 ± 0.45	0.94 ± 0.19	–6.94 ± 0.26	4.28 ± 0.39
60×60	RL	—	—	—	—
	FSA-MDP	—	—	—	—
	FSARS	0.09 ± 0.31	0.98 ± 0.07	–21.79 ± 1.87	10.27 ± 2.20

**Note:** The dash “-” signifies that the execution time surpassed the 3600-second threshold without yielding a solution.

For the above three scenarios, we verify the low and high levels of FSARS. The simulation results prove the efficiency of the proposed FSARS method. In details, FSARS can generate a safe path by reducing the conflicts with humans. The simulation results demonstrate that a conflict-free path within a time-horizon window can be obtained by FSARS. In conclusion, the proposed FSARS can find a safe path for the agent performing multiple tasks with high task success rate. It outperforms traditional methods such as RL, FSA-MDP, and MCTS. However, FSARS also cannot provide the guarantee that the planned path is completely conflict-free because we consider human-shared environments, where humans move randomly, which is a quite difficult problem. FSARS is able to generate a safe path within a longer time-horizon window, in which it is conflict-free from the view of the simulation results. Note that there is no theoretical guarantee is provided.

**Fig 19:**
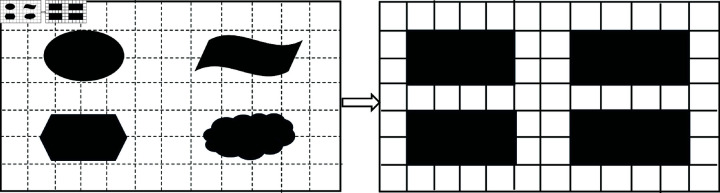
Grid map construction of irregular obstacles.

In this paper, we mainly consider the 2D grid environment where warehouses are usually well-form designed. The shelves in warehouses are regular. For the case of irregular obstacles, we can divide them and construct a grid map no matter what kind of irregular obstacles, as shown in Fig 19.

## 7 Conclusion and future work

In this study, we address path planning and task controlling problems simultaneously, that is, planning a global safe path for an agent conducting multiple tasks in human-shared environments. The most difficult case, in which humans move completely randomly, is considered in this study. We develop a mobile robot safe planner in which a double-layer FSARS performing safety-first search.

FSARS is unique in both low and high levels. The safe A^*^ method is extended in the low-level to generate a safe path considering stochastically moving humans. The proposed high-level of FSARS is an FSA-based graph search method, and it performs safety-first search to find an optimal safe path executing multiple tasks. Compared with the RL method, FSARS reduces the average conflict by 65.4% and improves the task success rate by 34.4%. The simulation results show the efficiency of our proposals in reducing conflicts and high task success rate. However, our algorithm still cannot provide safety guarantee theoretically.

Ongoing directions include the following: (1) more investigation on the calculation of uncertainties, (2) generation of a longer time-horizon window with conflict-free properties, and (3) study of the theoretical safety guarantee of generated paths.
